# CD133-positive cancer stem cells from Colo205 human colon adenocarcinoma cell line show resistance to chemotherapy and display a specific metabolomic profile

**DOI:** 10.18632/genesandcancer.23

**Published:** 2014-07

**Authors:** Zangiacomi Vincent, Kenichi Urakami, Koji Maruyama, Ken Yamaguchi, Masatoshi Kusuhara

**Affiliations:** ^1.^ Regional Resources Division, Shizuoka Cancer Center Research Institute, Shizuoka, Japan; ^2.^ Cancer Diagnostics Research Division, Shizuoka Cancer Center Research Institute, Shizuoka, Japan; ^3.^ Experimental Animal Facility, Shizuoka Cancer Center Research Institute, Shizuoka, Japan

**Keywords:** cancer stem cells, CD133, metabolomics, adenocarcinoma, CE-TOF-MS

## Abstract

During the past decade, cancer stem-like cells (CSCs) have drawn substantial interest in cancer research since they have been described as major targets to improve treatment of tumors and to prevent recurrence and metastasis. In this paper, we report on the search for CSCs within the Colo205 human adenocarcinoma cell line. We describe that CD133 (prominin) was the only reliable marker for the isolation and characterization of CSCs within a Colo205 cell population. CD133-positive cells displayed many CSC characteristics, such as tumorsphere formation ability, expression of early-stage development markers, high invasiveness, raised tumor initiation potential and resistance to cisplatin chemotherapy treatment. In vitro analyses also highlighted a specific metabolomic profile of CD133-positive cells and we concluded that the chemotherapy resistance of CSCs could be related to the quiescence of such cells associated with their reduced metabolism. Furthermore, in vivo metabolome analyses suggested that a high level of circulating glutathione molecules could also promote treatment resistance. From the perspective of metabolomics, we also discuss the controversial use of serum-free in vitro cultures for CSC enrichment prior to further phenotype characterization.

## INTRODUCTION

It is now well established that cancer stem-like cells (CSCs) can be identified within in vivo tumor bulks or in vitro cell cultures thanks to several markers [1, 2, 3]. Many studies have already reported the presence of CSCs in different solid tumors, such as breast, brain, prostate, lung, ovary, colon, pancreas, liver, melanoma, head and neck [4, 5, 6, 7]. The most common membrane markers used for the sorting or analysis of CSCs are CD133, CD44 and aldehyde dehydrogenase (ALDH1), while many other specific markers have also been described, such as CD24 and epithelial-specific antigen [3, 4, 8, 9, 10]. Nevertheless expression of these markers is highly heterogeneous, depending on cancer localization, cell type and the tumor microenvironment.

CD133 (prominin) is a membrane glycoprotein that was first described on hematopoietic and neural stem/progenitor cells. CD133 protein was later reported as a marker of poor prognosis within cancers (*e.g.* colorectal/breast cancer and myeloid leukemia) and was subsequently confirmed as being specifically expressed by the CSC population [3, 11, 12, 13, 14]. Another molecule, CD44, is expressed by a large number of mammalian cell types. This protein was first discovered on human hematopoietic stem cells and then identified in several cancers [4, 9]. Some studies also revealed that ALDH1, another common marker used for CSC identification, was also intimately correlated with tumorigenesis [1, 8, 15, 16].

Several studies have already reported the presence of CSCs within colon cancers; they were described as a rare population characterized by self-renewal capacity, clonogenicity, multipotency and chemoresistance [3, 5, 10, 17]. The scarcity of CSCs within cancer unfortunately impedes their detection and isolation. However, it has been well established that serum-free cultures can lead to in vitro stem cell enrichment through tumorsphere formation [6, 14].

Our study focused on the analysis of metabolome using capillary electrophoresis time-of-flight mass spectrometry (CE-TOF-MS). We characterized and quantified over 100 intracellular metabolites involved in human metabolic pathways. Several metabolomic approaches in cancer research have been reported yet [18, 19, 20, 21] and many proteomic applications for analyzing urine or serum of patients have also been conducted, confirming the high resolution and sensitivity of such techniques for clinical diagnoses [22, 23].

In this study, we highlighted that CD133 is the only reliable marker for CSC characterization within the Colo205 colon adenocarcinoma cell line. Besides, metabolome profiles further revealed that the serum-free expansion protocol commonly used for in vitro proliferation of progenitors may create too many artifacts in cell metabolism, reducing the efficacy of such a method prior to phenotype analyses or sorting.

## RESULTS

### Colon adenocarcinoma cell lines can form tumorspheres in vitro

**Figure 1:**
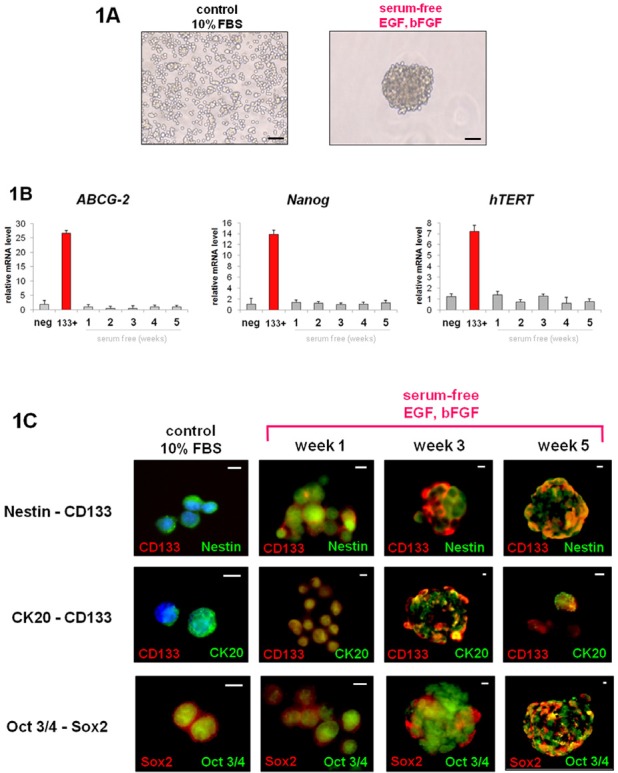
Serum-free cultures enrich Colo205 cells in CSCs A. Colo205 cells cultured in 10% FBS or serum-free conditions. Scale bar = 50 μm. B. Relative expression of ABCG-2, nanog and hTERT mRNA of Colo205 cells grown under 10% FBS conditions (control), CD133+ sorted cells and serum-free growing cells (week 1 to week 5). C. Immunofluorescence analyses of nestin, CD133, CK20 and Oct3/4 proteins. Images show 10% FBS Colo205 growing cells (control) and serum-free growing cultures (week 1 to week 5). Scale bar = 5 μm.

We compared the in vitro culture of cells in a basal condition (10% FBS) and in a serum-free condition. The cultures revealed that the Colo205 cell line could give rise to tumorspheres in serum-free conditions only. In contrast, cultures under FBS conditions only led to a layer of adherent confluent cells (Figure 1A). To rule out the possibility that cells may aggregate due to culture at a high concentration of cells, only 100 cells were seeded in each well. Tumorspheres could also be observed under these conditions. These results confirmed that tumorsphere-like colonies could be obtained from the Colo205 cell line and expanded in serum-free medium supplemented with EGF and bFGF, even under conditions with an extra-low cell concentration.

### In vitro characterization of Colo205 cell line

**Figure 2:**
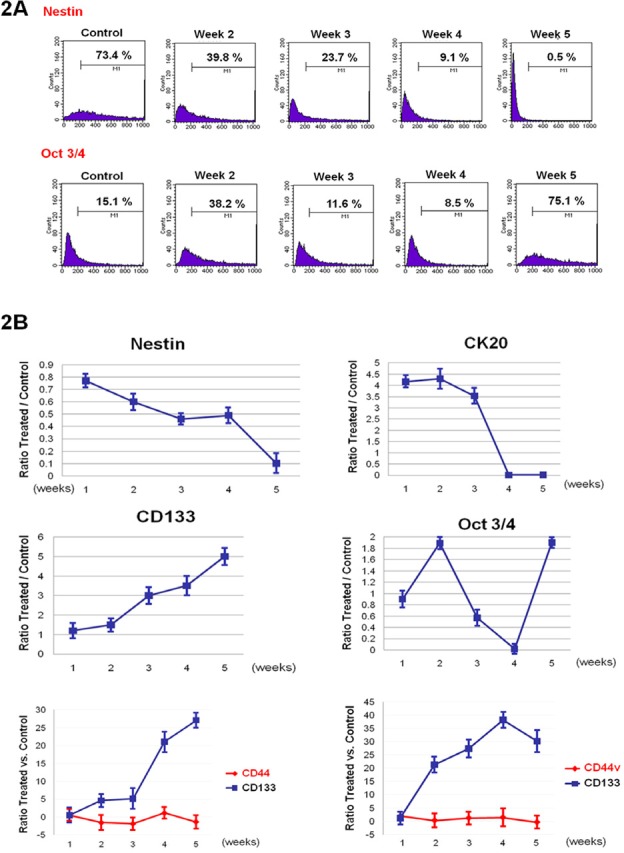
Serum-free cultures lead to the loss of early and late development markers and increase of stem-like markers A-B. Cytometry analyses of Nestin, CK20, CD133, Oct3/4, CD44 and CD44v9 on 10% FBS Colo205 growing cells (control) and serum-free growing cultures (week 1 to week 5).

As in vitro serum-free conditions could lead to floating cell enrichment and colonies, we decided to analyze the phenotype further. mRNA expression levels in Colo205 tumorspheres were not significantly different from those under basal conditions (FBS 10%), even after five weeks of culture, with regard to the expression of early-development CD133, hTERT and ABCG-2 mRNA (Figure 1B). Nevertheless, immunofluorescence and cytometry analyses showed an evolution of phenotype when cells were exposed to serum-free medium. The analyses confirmed the loss of early and late differentiation markers such as nestin and cytokeratin 20 (CK20), while the expression of embryonic and stem cell markers such as oct3/4 and CD133 was increased in non-serum cultures (by two and five times, respectively, compared with the control) (Figures 1C, 2A, 2B).

### CD133+ Colo205 cells exhibit CSC characteristics

**Figure 3:**
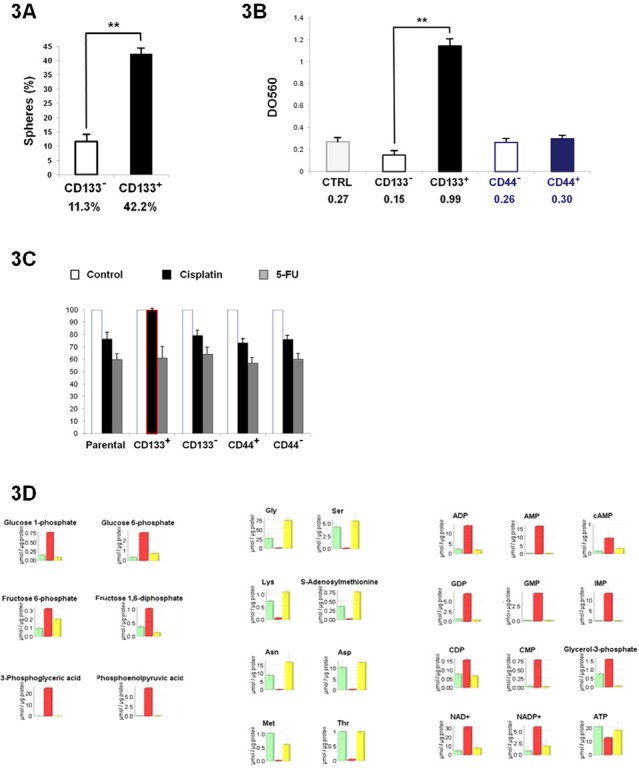
Colo205 CD133+ cells exhibit the phenotypic profile of CSCs A. Tumorsphere evaluation of CD133+ and CD133- sorted cells. B. Invasiveness of different cell fractions measured using a collagen-based invasion kit (relative DO measured at 560 nm). C. Survival assay after chemotherapy treatment with cisplatin and 5-FU. D. Metabolite quantification after CE-TOF-MS experiments. Only metabolites of interest for Colo205 cells cultured in 10% FBS (control; green bar), CD133+ sorted cells (red bar) and CD44+ sorted cells (yellow bar) are reported here.

To assess the stem-cell profile of different cell fractions, we further performed cell selection on the Colo205 cell line. RT-PCR analyses revealed that Colo205 CD133+ purified cells exhibited significantly increased expression of early-development mRNAs such as CD133, ABCG-2, hTERT, oct4, nanog and nestin (p<0.05) compared with basal Colo205 cells (neg.) (Figure 1B). We also investigated the colony formation of both CD133+ and CD133- sorted cells from the Colo205 cell line in soft agar. Our results showed that colony formation efficiency levels for CD133+ and CD133- cells were 42.2±2.3% and 11.3±3.1% respectively, indicating that the CD133+ population displayed high clonogenicity compared with the resulting CD133- fraction (Figure 3A).

### CD133+ population from Colo205 is enriched in ALDH1-positive cells

We investigated the presence of the ALDH1 marker on Colo205 cells after CD133 selection. Quantification was then performed by cytometry analyses. The results revealed that 1.7±0.2% of CD133+ cells co-expressed the ALDH1 molecule, while 0.2±0.1% of CD133- cells did. The Colo205 CD133+ cell population was thus shown to be enriched in terms of ALDH1-expressing cells.

### CD133+ subpopulation displays increased invasiveness.

In vitro invasion assays were performed on several Colo205 sorted subpopulations using a collagen-based cell invasion method. The results showed that the CD133+ purified population was significantly more invasive than the CD133- resulting fraction or the control fraction (absorbance units (AU)=0.99, 0.15 and 0.27, respectively; P<0.01). No significant difference was observed for both CD44+ and CD44- fractions (AU=0.30 and 0.26, respectively) (Figure 3B). These results confirmed that Colo205 CD133+ cells exhibited greater tumorigenic capacity than the CD133- and CD44+/− cells.

### CD133+ cells are resistant to cisplatin chemotherapy

To assess drug sensitivity, chemotherapy survival assays were performed after CD133 and CD44 selection. Cells were exposed to 10 μM cisplatin and 5-FU, and then analyzed for survival. The CD133+ cell population exhibited significantly increased resistance to cisplatin anti-tumor treatment, while no significant difference was observed concerning 5-FU drug treatment (Figure 3C). These findings suggested that the Colo205 CD133+ population has increased resistance to the common colon anti-cancer chemotherapeutic drug cisplatin, while other cell populations do not show any resistance to both of the investigated drugs.

### Colo205 CD133+ cells have specific metabolome expression

To identify specific metabolome features, CE-TOF-MS analyses were performed after cell sorting. The results showed that only the CD133+ purified fraction had a significantly different metabolome profile compared with the other cell fractions. No significant difference was observed for CD44+, CD44-, CD133- and parental Colo205 cells (Figure 3D). On the one hand, the CD133+ cell fraction exhibited high overall increases in glycogen, components of the citrate cycle, nucleotides and components of co-factor metabolism pathways (from two to ten times higher than in parental Colo205 cells). On the other hand, amino acid metabolites were barely detectable within CD133+ cells compared with those in other fractions. CE-TOF-MS analyses revealed that 28 metabolites were upregulated while 53 metabolites were downregulated in CD133-positive cells (Figure S1). These metabolome results reinforced the exclusiveness of the phenotype observed within the CD133+ cell population derived from the Colo205 cell line.

### Serum-free cultures lead to restructuring of the whole metabolome

As phenotypic differences had been highlighted among cultured Colo205 cells, we performed CE-TOF-MS on these cells. The results indicated that serum-free culture (for three to five weeks) led to overall increases in the metabolome related to glycogenosis, glycolysis, the citrate cycle, amino acid synthesis and nucleotide metabolism pathways. Compared with the control, 78 metabolites were upregulated while only five metabolites were downregulated after five weeks in serum-free culture (Figure S2).

### CD133+ cells have a slow development rate in vitro

**Figure 4:**
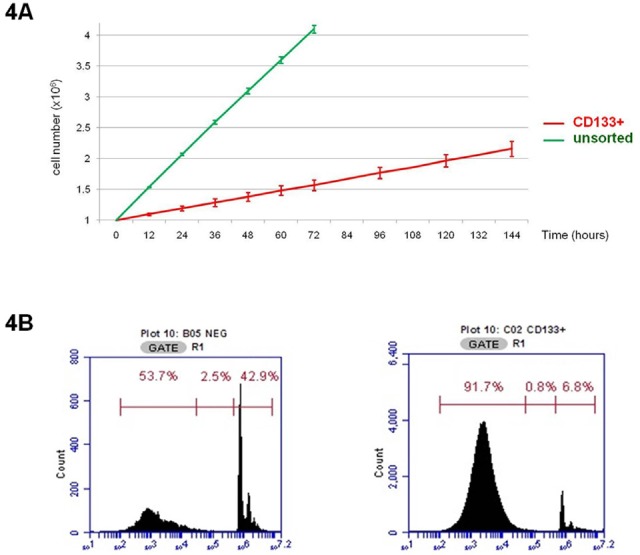
Growth evaluation of CD133+ cells derived from Colo205 A. Doubling time calculation of unsorted Colo205 cells vs. CD133+ sorted cells. B. Cell cycle analysis (PI staining) of Colo205 CD133- and CD133+ sorted cells.

We investigated the growth of unsorted Colo205, CD133+ and CD133- cell fractions in basal 10% FBS in vitro culture. While unsorted parental Colo205 cells had a population doubling time of less than 24 h (22±1.4 h), CD133+ cells had a far slower development rate (126±8.7 h) (Figure 4A). Cell cycle analyses revealed that CD133+ cells had a decreased transition rate out of the G_0_/G_1_ phase into the S phase. In fact, less than 10% of CD133+ cells were in the S phase (6.9±0.7%), while most of them remained in the G_0_/G_1_ phase (82±2.1%). In contrast, almost half of CD133- cells remained in the G_0_/G_1_ phase (51.2±2.3%) or had started the S phase (43.1±1.6%) (Figure 4B).

### CD133+ cells are more tumorigenic in vivo

**Figure 5:**
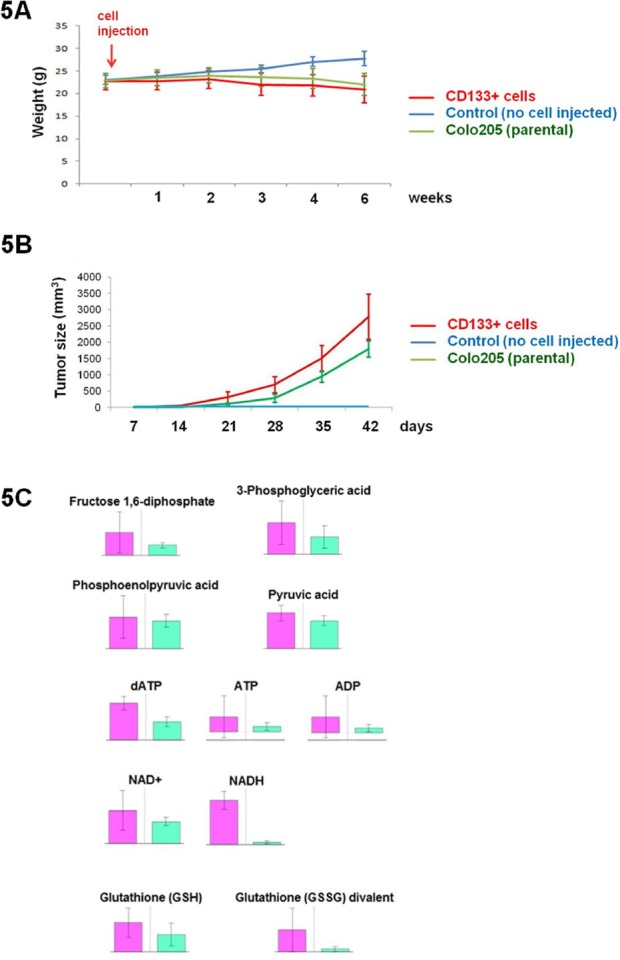
CD133+ cells are more tumorigenic in vivo than unsorted Colo205 cells A. Evaluation of mouse weight after cell inoculation. B. Tumor size evaluation after inoculations of parental Colo205 cells (unsorted) and CD133+ cells in Balb/c mice. C. Metabolite quantification after CE-TOF-MS experiments. Metabolites were quantified from mouse serum after tumor development from Colo205 CD133+ sorted cells (purple bar) and parental unsorted Colo205 cells (green bar). Metabolites of interest reported here were quantified six weeks after s.c. inoculation.

To assess the in vivo tumorigenicity of CD133+ cells, various Colo205 cell fractions were injected into mice at different concentrations. *BALB/c Nude* mice (n=6) were subcutaneously (s.c.) inoculated with parental cells, or purified CD133+ or CD133- Colo205 cells. Tumor development was more efficient for CD133+ purified cells than for the other cell fractions (Figures 5A, 5B). While only 5×10^4^ CD133+ cells were shown to be able to expand and give rise to a complete tumor bulk after seven days (2/6), it took more than six weeks to observe formation of a single tumor bulk from 2×10^6^ CD133- purified inoculated cells (1/6) (Table S1).

As tumor growth from purified CD133+ cells was greater than from CD133- cells under identical conditions, secondary tumors were also found in a few mice inoculated with purified CD133+ cells only (2/12), suggesting increased metastatic potential for this cell population. No metastasis was found in “CD133-negative” and “parental” inoculated mice after 10 weeks. We also observed that 30% of the mice inoculated with CD133+ purified cells suddenly died in less than five weeks (3/10), independently of tumor size. Meanwhile, in the CD133- group, no mice died, even 12 weeks after s.c. inoculation (0/10; data not shown). These findings confirmed that Colo205 CD133+ cells have higher tumorigenicity than other cell populations.

### Tumor development of Colo205 CD133+ cells led to reorganization of metabolome expression in mice

Cytometry analyses were performed on each sample to confirm the selection efficiency prior to inoculation. CD133+ purity was higher than 97% after sorting and no CD133+ cells were detectable in the remaining CD133- cell fraction (n=12). On the basis of our preliminary in vivo tumor growth development results, we chose to inject 2×10^5^ CD133+ cells, while 1×10^7^ cells were injected in the CD133-/parental group, so we could compare metabolite variations under similar tumor growth conditions at each stage of our study. Cytometry analyses first revealed that CD133+ purified cells gave rise to a heterogeneous population in vivo, mainly composed of CD133- cells. In fact, six weeks after inoculation of purified CD133+ cells, only 2.6% of remaining CD133+ cells could be detected within tumor bulks (2.6±0.7%; n=6). On the other hand, while no CD133+ cells could be detected in the CD133- fraction prior to injection, a few CD133+ cells were then detected in tumors after six weeks (0.29±0.15%; n=6). Thus, even if the CD133+ cell population was drastically reduced during the tumor development process, prominin-1 expression in tumors was still eight times higher in the CD133+ group than in tumors derived from the CD133- fraction (2.6% vs. 0.29%, respectively; n=6) (Table S2).

### Metabolome analyses were performed once a week on each mouse following s.c. injection

Three mouse groups were thus analyzed: those with “*parental*” cells containing unsorted Colo205 cells, and those with “*CD133+*” and “*CD133-*” sorted populations. Despite precise CE-TOF-MS analyses (n=90), only limited variation in metabolite concentrations was highlighted among the three different groups tested. Specifically, metabolome analyses revealed that only components of the glycolysis pathway, dATP, ATP, ADP, NAD+, NADH and glutathione (mono-GSH and divalent γ-GCS forms) molecules, were significantly overexpressed in the serum of mice that developed tumors from CD133+ purified cells compared with those in tumors derived from the CD133- population (Figure 5C). These results confirm the specific features of the CD133+ CSC population within the Colo205 adenocarcinoma cell line.

## DISCUSSION

Tumor tissues have been shown to contain highly heterogeneous cell populations [2, 9, 17]. However, the role of each population and their interrelationships remain unclear and need to be determined to establish alternative effective diagnostic methods and therapeutics. Even though the existence of CSCs remains controversial, some authors have argued that new revolutionary therapeutic approaches could be achieved by focusing on stem-like cells subpopulation [18, 20]. Indeed, CSCs were shown to have high clonogenicity and increased invasiveness, being able to give rise to a complete tumor bulk and to enhance metastasis, even from a few cells remaining after surgical resection [24, 25]. Moreover, these cells have been shown to be more resistant to common chemotherapy and radiotherapy treatments in many solid tumors [14, 26]. Thus, we postulate that eradicating such resistant cells could lead to a loss of clonogenicity and drastically reduce the risk of recurrence or metastasis after resection and treatment.

Previous studies have already described many markers to characterize CSCs [1, 9, 17]. Nevertheless, the presence of a universal marker for colon CSC characterization remains controversial. We demonstrated here that CD133 was the only reliable marker for phenotypic characterization of CSCs within the Colo205 cell line. In fact, the small population of CD133+ cells (1%–2%) exhibited both in vitro and in vivo characteristics of CSCs, such as high clonogenicity, increased invasiveness and high expression of specific stem cell markers (*i.e.* MAP2, nanog, oct3/4). Moreover, we highlighted that Colo205 CD133+ cells were more resistant to the anti-tumor drug cisplatin. Our results also showed high tumorigenic and metastatic potential of the CD133+ population after inoculation in mice. Even though the CD44v8-10 variant form was previously shown to be related to CSCs in stomach cancer, no relationship could be clearly established between CD133 and CD44 variant populations within our Colo205 colon cancer model [9, 27]. This reinforces the apparent absence of a common universal surface marker for CSCs.

Several metabolome analyses have recently been conducted in the field of cancer research to discover new diagnostic markers or potential therapeutic targets [18, 19, 23, 28]. The high resolution and sensitivity of this technique combined with molecular analyses revealed an additional powerful tool for advanced research. Our results highlighted that Colo205 CD133+ cells that harbor a stem-cell-like phenotype also have a specific metabolome profile. CD133+ cells exhibited increased expression of components related to the glycolysis pathway, the citrate cycle pathway and co-factors. Moreover, CD133+ cells exhibited a drastically increased level of nucleoside mono- and diphosphate molecules (*e.g.* ADP, cAMP, AMP), while other nucleotides like ATP were slightly decreased compared with those in CD133-negative cells. Additionally, amino acid content almost completely disappeared in CD133+ cells, suggesting that protein synthesis was drastically inhibited in CSCs. Cellular differentiation has been shown to be associated with pronounced downregulation of glycolysis [14, 21, 29, 30]. Thus, overexpression of this metabolic pathway in CD133+ cells can be considered to be suggestive of the “non-proliferative” state of this population. Moreover, acetyl-CoA, which is required for chromatin acetylation, cholesterol and glucose-dependent lipid synthesis, was completely inhibited in CD133+ cells, in contrast to that in the control. This specific metabolomic profile and the fact that most (>90%) CD133+ cells remained in the G_0_/G_1_ phase confirm the hypothesis supported by previous studies, namely, that CSCs have a propensity to remain in a relatively quiescent state [31, 32]. We postulate here that the accumulation of mono- and diphosphate nucleosides associated with strong down-regulation of amino acid content could be associated with an energy-saving process. Indeed, cAMP, AMP and ADP can be easily converted to ATP via aerobic respiration in the mitochondria [33]. This “pre-ATP” stock may be a versatile energy source for amino acid synthesis, which is known as a strongly endergonic process [34]. Thus, the conditions required for entry into the G_2_/S phase seem to be possible if the proliferation of CD133+ cells is needed.

In our study, CD133+ cells were shown to be resistant to cisplatin. While several mechanisms of cisplatin resistance have already been described, including changes in cellular uptake and drug efflux, increased drug detoxification, inhibition of apoptosis and increased DNA repair [26, 32, 35], we postulate here that the metabolic quiescence and slow proliferation of CD133+ cells could also provide another opportunity for CSCs to escape from antitumor chemotherapy treatments. As most CD133+ cells stay in the G_0_/G_1_ phase, a strained activation of all CSCs to differentiate could be an interesting potential therapy to explore, promoting the subsequent targeting of cells by chemotherapy drugs that are specifically aimed to affect cell division [36].

In vivo analyses also confirmed the existence of a specific metabolic profile when mice were inoculated with purified CD133+ cells. We reported a significant increase of components of the glycolysis pathway, glutathione molecules, NAD+/NADH and ATP in mouse serum after tumor development. Besides overexpression of GSH and γ-GSH molecules, which has been shown to enhance cisplatin resistance, a high concentration of circulating ATP and co-factors could also promote tumor cell proliferation [32, 35]. This specific metabolome observed in mice indicated that a high concentration of CD133+ cells in a tumor might lead to a strong Warburg effect in vivo [37, 38, 39]. As an increased rate of metastasis was observed after CD133+ cell inoculation, rearrangements of the cellular micro-environment could also lead to CD133+ progenitors circulating and enhance the development of metastasis [21, 24, 40, 41, 42].

Our in vitro studies also highlighted that the metabolome profile was unnaturally elevated in tumorspheres compared with that in control cells. Almost 100 molecules were regulated in opposite ways depending on the method used for culture (*i.e.* 10% FBS vs. serum-free) and important variations were also observed in mRNA and protein levels between CD133+ purified cells and tumorsphere-derived ones. Even though tumorsphere culture is the best way to expand and enrich CD133+ cells in vitro, we accept that the serum-free expansion protocol conventionally used for stem/progenitor cell proliferation is not reliable for use prior to CSC molecular analyses [43].

In this work, we confirmed that Colo205 tumor bulks consisted of a heterogeneous population in which two clear cell subpopulations are maintained [44, 45]. On the one hand, there is the CD133+ cell population, which is a small population with clear CSC characteristics: resistance to chemotherapy, slow development rate, low metabolism, strong capacity for tumor formation, tissue invasion and metastasis formation [3, 13, 26, 46]. On the other hand, the remaining CD133- cells, with a rapid proliferation cycle, could barely give rise to tumors in vivo. Despite that, accurate discrimination of the stem cell subpopulations remains a difficult process. Indeed, some CD133+ cells are barely detectable as the externalization of the CD133/prominin marker on the membrane has not yet been completed at the time of the sorting/analysis process [47]. Thus, a few cells expressing CD133/prominin but displaying a false-negative phenotype cannot be detected or isolated by conventional cytometry/sorting techniques, and might contaminate the pure CD133- cell fraction. This could explain why a few CD133+ cells could be detected after the development of a tumor derived from the purified CD133- population. Even though the need for collaboration between stem and non-stem cell subpopulations for efficient proliferation/differentiation processes in different models has already been described [17, 48, 49, 50], interactions among CD133+ cells, CD133- cells and the tumor environment remain unclear [17]. If the differentiation of CD133+ cells giving rise to CD133- cells is proven, we also support the hypothesis that, in the absence of CD133+ cells, some CD133- cells might dedifferentiate and give rise to CD133+ CSCs able to promote tumor development and metastasis. Unfortunately, given the probable rarity of this and its occurrence only under specific in vivo conditions, spontaneous dedifferentiation is assumed to be a complicated process to observe and a major challenge to identify in future studies.

## METHODS

### Cell lines and in vitro cultures

The Colo205 human adenocarcinoma colon cell line was obtained from the American Type Culture Collection (ATCC). Cells were cultured in RPMI 1640 medium supplemented with 10% fetal bovine serum (FBS, Gibco). Tumorsphere formation was performed in serum-free medium supplemented with 20 ng/mL human recombinant EGF (PeproTech) and 10 ng/mL human recombinant bFGF (PeproTech).

### CD133+ and CD44+ cell selection

Colo205 cells were collected using collagenase (Sigma-Aldrich) and were then fractionated using a CD133 or CD44 cell isolation kit (Miltenyi Biotec). Magnetic sorting was performed at least twice for each sample. The purity of sorted cells was evaluated by flow cytometry using FACSCalibur (BD Biosciences) after labeling with anti-human CD133/2 or CD44 antibody (Miltenyi Biotec).

### Immunofluorescent staining

For intracellular staining, cells were counted, washed twice in phosphate-buffered saline (PBS, Gibco) and fixed in PBS/3.7% formaldehyde (Sigma-Aldrich) for 20 minutes at 4°C. The cells were then permeabilized with PBS/0.1% Triton X-100 (Sigma-Aldrich) for 10 minutes at room temperature. They were subsequently washed and incubated for one hour at 4°C with mouse monoclonal primary antibodies: anti-nestin (MAB1259, 5 μg/mL; R&D Systems), anti-CK20 (M7019, 1/200; Dako) and anti-sox2 (MAB2018, 5 μg/mL; R&D Systems), or with rat monoclonal primary antibody anti-oct3/4 (MAB1759, 5 μg/mL; R&D Systems). After several washes, cells were incubated for 30 minutes with the appropriate secondary antibody: anti-mouse antibody conjugated to fluorescein isothiocyanate (FITC) (115-096-062, 1/200; Jackson Immunoresearch), PE-conjugated anti-mouse antibody (115-116-068, 1/200; Jackson Immunoresearch), rhodamine-conjugated anti-mouse antibody (115-026-003, 1/200; Jackson Immunoresearch) or FITC-conjugated anti-rat antibody (112-096-003, 1/200; Jackson Immunoresearch). For membrane staining, cells were incubated for 30 minutes at 4°C with anti-CD133-PE (130-090-853, 1/200, Miltenyi Biotec), anti-CD44-PE (130-095-180, 1/200, Miltenyi Biotec) or primary rat monoclonal antibody anti-human CD44v8-10 (ALG011, 1/1000, Link Genomics). For anti-CD44v8-10 detection, cells were further stained with secondary FITC-conjugated anti-rat antibody (112-096-003, 1/200; Jackson Immunoresearch). Nuclear DNA staining was also performed with 4',6'-diamidino-2-phenylindole (DAPI; D8417, 1/5000; Sigma-Aldrich). Cells were then washed and viewed under a fluorescent microscope. Negative controls with anti-human IgG antibodies were used to discard false-positive cells in the immunofluorescent staining (Jackson Immunoresearch). Aldefluor staining was also performed to quantify ALDH1-positive cells (Aldagen).

### Flow cytometry analysis

To assess the sorting process of the Colo205 cell line, selected cells were stained by both relevant monoclonal antibodies: PE-conjugated AC133/2-PE antibody (130-090-853, 1/200; Miltenyi Biotec) and PE-conjugated CD44-PE antibody (130-095-180, 1/200; Miltenyi Biotec), or with similarly conjugated isotype-matched antibodies, for 30 minutes at 4°C. Cytometry analyses revealed that 95.2±2.9% (n=19) of the selected cells were CD133+ after sorting, while 94.3±3.6% (n=15) of the selected cells were CD44+ after sorting. Furthermore, no CD133+ or CD44+ cells could be detected within the respective remaining negative fractions (*i.e.* CD133- or CD44-).

### Reverse-transcription polymerase chain reaction (RT-PCR)

Total RNA was isolated using Trizol Reagent (Invitrogen), according to the manufacturer's procedures. Primers were synthesized by Sigma-Aldrich. The specific oligonucleotide primers for the GAPDH gene were: TGA AGG TCG GAG TCA ACG GAT TTG G (sense) and CAT GTA GGC CAT GAG GTC CAC CAC (antisense), for the nestin gene: AGG ATG TGG AGG TAG TGA GA (sense) and TGG AGA TCT CAG TGG CTC TT (antisense), for the CD133 gene: TTA CGG CAC TCT TCA CCT (sense) and TAT TCC ACA AGC AGC AAA (antisense), for the oct4 gene: CGC ACC ACT GGC ATT GTC AT (sense) and TTC TCC TTG ATG TCA CGC AC (antisense), for the ABCG2 gene: CTG AGA TCC TGA GCC TTT GG (sense) and TGC CCA TCA CAA CAT CAT CT (antisense), for the Nanog gene: AAT ACC TCA GCC TCC AGC AGA TG (sense) and CTG CGT CAC ACC ATT GCT ATT CT (antisense), and for the hTERT gene: AGC CAG TCT CAC CTT CAA CCG C (sense) and GGA GTA GCA GAG GGA GGC CG (antisense). RNA was quantified with the Qubit RNA BR Assay Kit (Invitrogen). cDNA was synthesized using Prime Script 1^st^ strand cDNA Synthesis Kit (Takara Bio). The PCR reaction mixture contained 5 μL (20 μM) of the above specific primers, 5 μL of Taq DNA Polymerase, 16 μL of 4×dNTP, 20 μL of 10× buffer, 20 μL of cDNA and 133 μL of ddH_2_O. The conditions used were as follows: denaturation at 95°C for 5 min; 30 cycles of annealing at 65°C (for GAPDH and hTERT), 63°C (for oct4 and nanog), 60°C (for ABCG2), 56°C (for nestin) or 54°C (for CD133) for 15 s, and extension at 72°C for 1 min; and then heating at 72°C for 7 min. The PCR products were resolved on a 2% agarose gel containing fluorescent nucleic acid gel stain GelRed™ (Biotium). Acquisition of gel pictures and quantification were then performed with ImageQuant TL 7.0 (GE Healthcare).

### Colony formation assay

Colony formation assay was performed in soft agar. A base layer was prepared by mixing 1% soft agar (Invitrogen) in the medium. Then, cells were suspended in growth medium containing 0.3% soft agar and seeded upon the base layer at a density of 2500 cells per well. All experiments were conducted at least in triplicate. Plates were maintained at 37°C in a humidified 5% CO_2_ incubator and medium was added every three days. After three weeks, colonies (>10 cells) were counted under a microscope.

### Cell invasion assay

QCM Collagen-based Cell Invasion Assay Kit (Chemicon, Millipore) was used following the manufacturer's procedures. Cells were seeded into the upper insert at 1×10^5^ cells per insert in serum-free medium. Outer wells were filled with RPMI medium containing 10% FBS as a chemoattractant. Cells were then incubated for 48 hours. Non-invading cells were removed by swabbing the top layer of collagen and cells able to migrate through the gel insert to the lower surface of the membrane were stained, solubilized and quantified by colorimetric measurements at 560 nm (Glomax Multidetector System, Promega). All experiments were conducted in triplicate.

### In vitro drug sensitivity assays

Drug sensitivity was evaluated using cisplatin and 5-fluorouracil (5-FU) (Sigma-Aldrich). In accordance with the manufacturer's instructions, cells were exposed to 10 μM cisplatin or 10 μM 5-FU for 72 h. Viability was then evaluated using the CellTiter 96 AQueous proliferation assay kit, by measuring absorbance at 490 nm (Promega). Experiments were conducted in triplicate for each sample.

### Doubling time calculation

Cells were plated at 10^5^ cells/well in six-well plates and manually counted with a hemocytometer. Doubling time (Td) was calculated using the following equation: Td=(t2-t1)×log(2)/log(q1/q2), where q1 and q2 represent the numbers of cells at times t1 and t2, respectively (n=6).

### Cell cycle analysis

The cell cycle was evaluated by cytometry using propidium iodide (PI) solution (Sigma-Aldrich). After fixation in 70% ethanol solution, cells were washed twice in PBS and stained using 250 μl of RNase solution (2 mg/ml, Sigma-Aldrich) added to 250 μl of PI solution (0.1 mg/ml in 0.6% Triton-X in PBS) for 45 minutes in the dark at room temperature. Cells were then transferred through capped tubes to avoid clumps during fluorescence detection. Samples were kept on ice and protected from light until cytometry analysis (Accuri C6, BD Biosciences).

### In vivo analyses

Male BALB/c mice (all six weeks of age) were obtained from the National Cancer Institute (Frederick, MD, USA). The mice were subcutaneously (s.c.) injected with 1×10^7^ Colo205 cells. CD133+ and CD133- cells were also injected after sorting (5×10^4^ to 2×10^6^ cells). At specified times after tumor inoculation, the mice were euthanized in a CO_2_ chamber and tumor cell suspensions were prepared from solid tumors by enzymatic digestion as follows. Minced tissues (<1 mm^3^) from tumors were incubated at 37°C for 90 minutes in standard medium containing 2% FBS, 50 U/ml collagenase I, 100 U/ml collagenase IV, 200 U/ml DNase-I and 2.5 U/ml protease XIV. Cells were then harvested for viability and immunocytometry characterization.

### Capillary electrophoresis–time of flight–mass spectrometry analyses (CE-TOF-MS)

Metabolite standards, instrumentation and CE-TOF-MS conditions. Instrumentation and CE-TOF-MS conditions followed the Human Metabolome Technologies guidelines (HMT Inc.). CE-TOF-MS experiments were performed on an Agilent capillary electrophoresis system coupled to an Agilent 6224 CE/TOF-MS analyzer (Agilent Technologies). System control and data acquisition were processed using the Agilent Chemstation software.

CE-TOF-MS was conducted in both positive and negative ion modes for each sample. Molecule separation was processed in fused-silica capillaries filled with 1 M formic acid as a background electrolyte. Samples were injected at 50 mbar, and a voltage of 27 kV (for cation mode) or 30 kV (for anion mode) was applied. Capillary temperature was maintained at 20°C while sample tray temperature was kept below 10°C. Sheath liquid was delivered at 10 μL/min. Capillary voltage was set at 4 kV for cation mode and 3.5 kV for anion mode; the flow rate of nitrogen gas (heater temperature 300°C) was set at 0.35 bar. Fragmentor, skimmer and Oct RFV voltages were set according to HMT setting recommendations (HMT Inc.). Exact mass data were acquired at a rate of 1.5 cycles per second over the range of 50–1000 *m/z*.

### Processing of CE-TOF-MS data

Raw data were extracted with MassHunter software (Agilent Technologies). Data processing was then performed using MasterHands software developed by HMT (MasterHands v2.8.0.3, HMT Inc.). Data analysis included noise-filtering, baseline correction, peak detection and integration of the peak area from sliced electropherograms (width 0.02 *m/z)*. Alignment was carried out and accurate *m/z* values were determined for each detected peak. All peak areas were then quantified by comparison to the values of internal standard molecules (relative area) to normalize signal intensities among multiple measurements. Undetected peaks with a threshold signal-to-noise ratio of 2 were given a peak area of 0 and then discarded.

### Metabolite identification

To discriminate metabolites of interest, peak identification was carried out based on matched *m/z* values and normalized migration times of standard compounds (standard mixtures H3301-10024, HMT Inc.). After alignment, all data were processed with MasterHands software to confirm and refine the surface of each peak according to the *m/z* values.

### Mapping process

Statistical analysis was carried out by calculating mean error and standard deviation for each group. Results were finally processed with Vanted software (V1.9) to create metabolic pathway mapping, including all metabolites of interest.

## SUPPLEMENTAL FIGURES AND TABLE


