# Supplementary material for: CD133-positive cancer stem cells from Colo205 human colon adenocarcinoma cell line show resistance to chemotherapy and display a specific metabolomic profile
Source: Genes Cancer. 2014 Jul;5(7-8):250–60. doi: 10.18632/genesandcancer.23 (PMC4162140; doi:10.18632/genesandcancer.23)
Supplement: Supplementary file 1 [file ganc-05-250-s001.pdf]

**CD133-positive cancer stem cells from colo205 human colon adenocarcinoma cell line show resistance to chemotherapy and display a specific metabolomic profile**

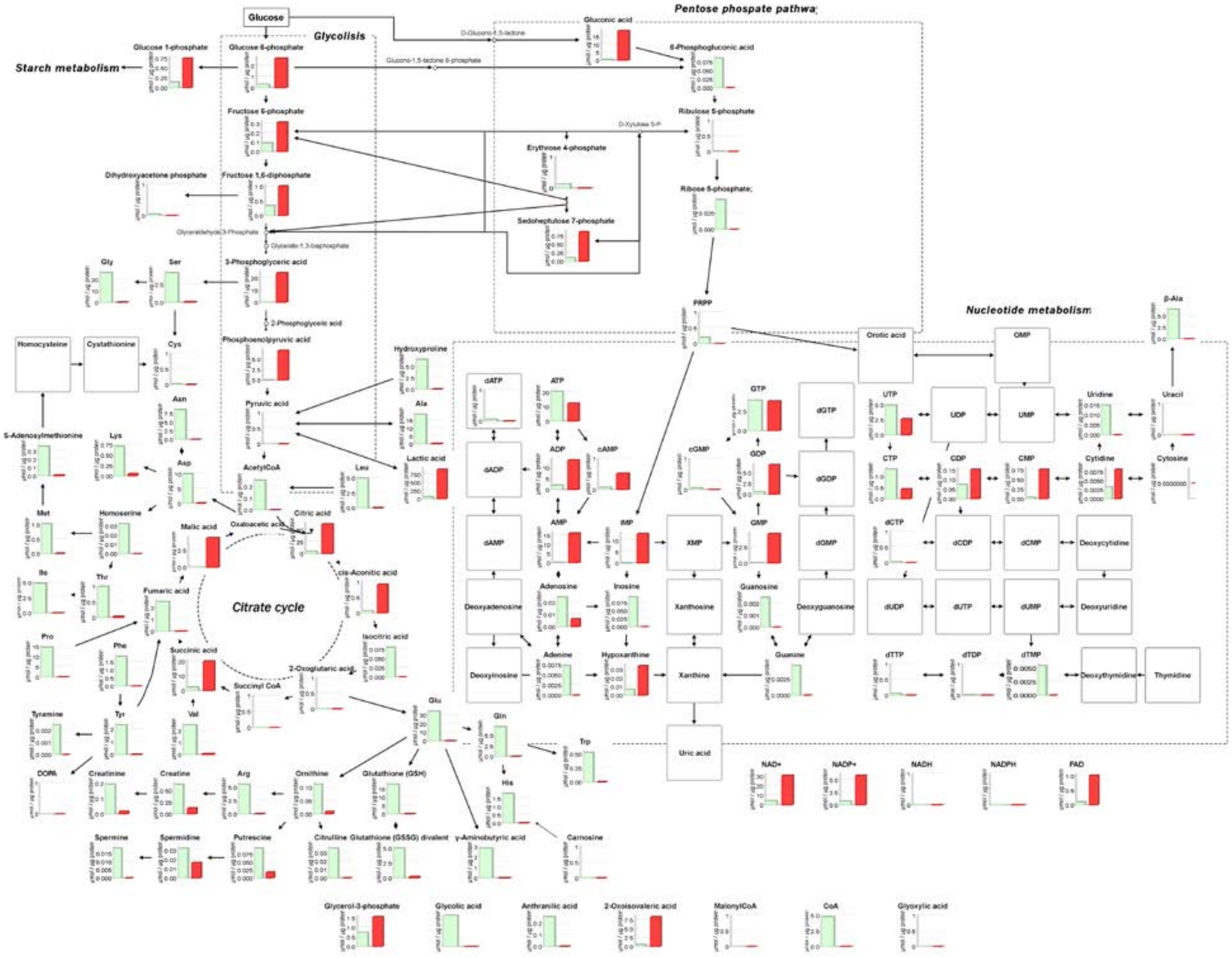


**Supplemental Figure 1: CE-TOF-MS mapping of Colo205 parental cells and CD133+ purified cells.** Green bars represent Colo205 parental cell metabolites, while red bars represent Colo205 CD133+ cell metabolites.

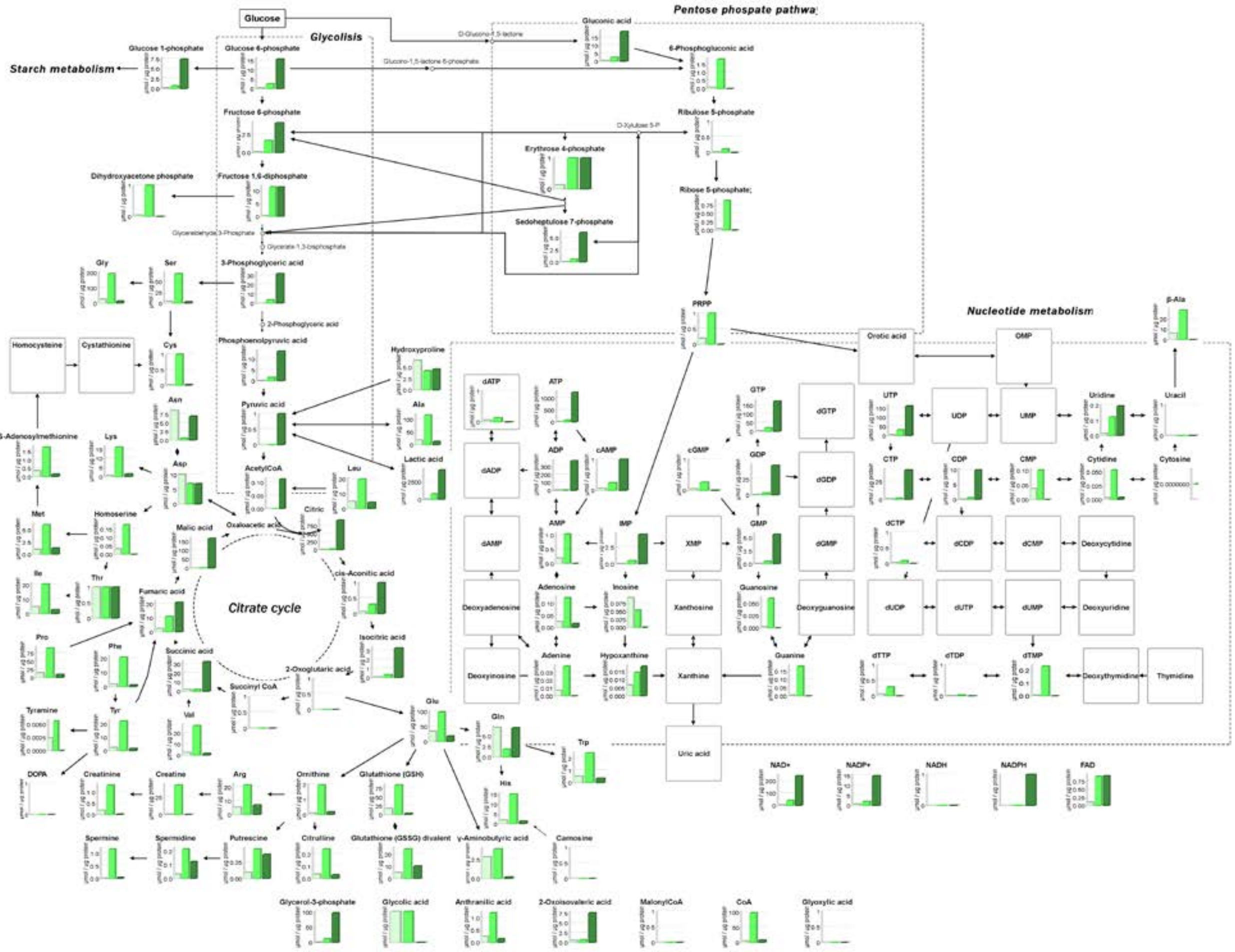


**Supplemental Figure 2: CE-TOF-MS mapping of Colo205 tumorspheres grown in serum-free conditions.** Results show Colo205 cells before culture (white green), and after three weeks (light green) and five weeks (green) of culture in serum-free conditions.

**Table S1.**
**Tumor-initiating ability of CD133+ versus CD133− fractions from Colo205 colon adenocarcinoma cell line**

| Colo205 | CD133+ cells | | | CD133− cells | | |
|---|---|---|---|---|---|---|
| | $5 \times 10^4$ | $1 \times 10^5$ | $2 \times 10^5$ | $5 \times 10^4$ | $1 \times 10^6$ | $2 \times 10^6$ |
| Data indicate the development of cancer nodules after subcutaneous injection of the indicated number of CD133+ and CD133− cells in *Balb c/c* mice. | | | | | | |
| **cancer nodules** | 2 \| 6 | 3 \| 6 | 6 \| 6 | 0 \| 6 | 0 \| 6 | 1 \| 6 |

**Table S2.**
**Characterization of CD133+ and CD133− cell subpopulations before and after tumor development**

| | | **Day 0** (prior to inoculation) | **Day 40** |
|---|---|---|---|
| **CD133+ sorting** | **CD133+** | 97.1±0.3% | 2.6±0.7% |
| | **CD133−** | 2.8±0.6% | 97.2±0.5% |
| **CD133− sorting** | **CD133+** | --- (no cell detected) | 0.29±0.15% |
| | **CD133−** | 100% | 99.7±0.2% |
